# Relationship Between Radiographic and Pathological Portal Vein‐Superior Mesenteric Vein Involvement in Neoadjuvant Treatment for Pancreatic Cancer: A Comparative Study of Neoadjuvant Chemotherapy and Chemoradiotherapy

**DOI:** 10.1002/wjs.70395

**Published:** 2026-05-07

**Authors:** Naoko Sekiguchi, Hidenori Takahashi, Hirofumi Akita, Kazuki Sasaki, Shinichiro Hasegawa, Yoshifumi Iwagami, Daisaku Yamada, Yoshito Tomimaru, Tadafumi Asaoka, Takehiro Noda, Junzo Shimizu, Shogo Kobayashi, Yuichiro Doki, Hidetoshi Eguchi

**Affiliations:** ^1^ Department of Gastroenterological Surgery Graduate School of Medicine the University of Osaka Suita City Osaka Japan

**Keywords:** NAT, pancreatic cancer, pathological PV‐SMV invasion, prognosis, radiographic PV‐SMV involvement

## Abstract

**Background:**

The relationship between the radiographic portal vein‐superior mesenteric vein (PV‐SMV) involvement and pathological PV‐SMV (pPV) invasion has been established in upfront surgery cases of pancreatic cancer (PC); however, evidence remains limited for patients receiving neoadjuvant therapy (NAT). This study aimed to evaluate the association of radiographic findings with pPV invasion in patients treated with NAT, and to examine whether this association differs between patients treated with neoadjuvant chemotherapy (NAC) and neoadjuvant chemoradiotherapy (NACRT).

**Methods:**

We retrospectively analyzed patients with PC whose tumors contacted the PV‐SMV on radiographic imaging before or after NAT. The relationship between radiographic findings and pPV invasion was evaluated in subgroups defined by pre‐ and post‐NAT imaging findings.

**Results:**

Tumor size, PV‐SMV contact length, and contact angle were significantly reduced in post‐NAT imaging. Radiographic PV‐SMV involvement showed limited association with pPV invasion in the entire cohort. The overall pPV invasion rate was 15% and did not differ between the NAC and NACRT groups (17% vs. 13%, *p* = 0.790). Among patients with a pre‐NAT tumor size of < 20 mm, the pPV invasion rate was significantly lower in the NACRT group than in the NAC group (29% vs. 0%, *p* = 0.007). This difference was not observed in patients with a pre‐NAT tumor size ≥ 20 mm (12% vs. 19%, *p* = 0.438). Across other radiographic classifications, pPV invasion rates remained comparable between treatment groups.

**Conclusions:**

The association of radiographic findings with pPV invasion differs between patients treated with NAC and NACRT in patients with small tumors.

## Graphical abstract

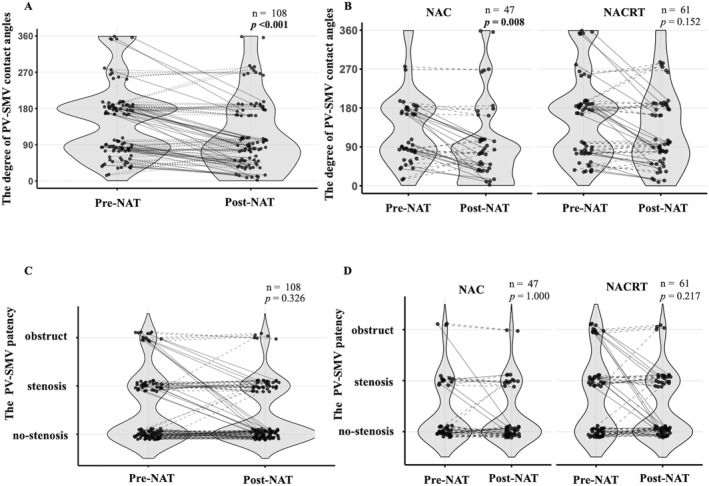

## 1 Introduction

Neoadjuvant therapy (NAT) is a standard treatment for pancreatic cancer (PC) [1, 2, 3]. Neoadjuvant chemotherapy (NAC) and neoadjuvant chemoradiotherapy (NACRT) have been used as NAT for PC. Accurate assessment of the response to preoperative treatment represents a critical component of NAT strategies for PC; in particular, appropriate evaluation prior to resection following NAT is essential for optimizing individualized treatment strategies [3]. However, treatment response after NAT is often difficult to evaluate radiographically because therapy‐induced fibrosis and inflammatory changes may persist around major vessels despite tumor regression [4].

Surgical resection with microscopically negative margins (R0 resection) remains the only potentially curative treatment for patients with PC [5]. Due to the anatomical characteristics of PC, tumors frequently contact the portal vein‐superior mesenteric vein (PV‐SMV) and require portal vein resection (PVR) for R0 resection in some cases, with 28%–69% of patients reported to undergo PVR [6, 7, 8, 9, 10, 11, 12]. However, the pathological PV‐SMV (pPV) invasion rate among patients undergoing PVR varies widely across studies, ranging from 41% to 91% [6, 7, 8, 9, 10, 11, 12]. While simple PV‐SMV resection and reconstruction is generally regarded as a safely feasible procedure [11], a subset of cases requires complex vascular reconstruction, such as those involving extensive longitudinal tumor infiltration necessitating interposition vascular grafting [13], or those with wide caudal extension of invasion requiring concomitant reconstruction of multiple jejunal veins [14]. In such cases, accurate preoperative prediction of pPV invasion could potentially allow the avoidance of unnecessary complex vascular resection or enable a simpler reconstruction procedure. Therefore, accurate preoperative assessment of true venous invasion is clinically important when determining the surgical strategy.

Several studies have previously investigated the relationship between radiographic PV‐SMV involvement and pPV invasion [11, 15]. Nakao et al. reported a relationship between the radiographic degree of PV‐SMV involvement on preoperative imaging and pPV invasion in an upfront surgery setting, where radiographic involvement correlated with both the incidence of PV wall invasion and postoperative prognosis [11]. Ishikawa et al. found that the diagnostic accuracy of pPV invasion based on the angiographic degree of involvement was 54%, and that invasion was underestimated in 40% of cases [15]. Importantly, these studies did not include patients who underwent NAT. Evidence has suggested that radiographic assessment after NAT may not accurately reflect pathological treatment response [3, 4]. Miyahara et al. reported a discrepancy between radiographic and pathological response evaluation following NAT for PC, and notably, this discrepancy was more pronounced in patients treated with NACRT compared to those treated with NAC [4]. Therefore, unlike previous reports in the upfront surgery setting, whether radiographic findings accurately reflect pPV invasion in patients treated with NAT remains unclear; furthermore, whether this relationship differs between NAC and NACRT is even less well established. Therefore, this study aimed to investigate the relationship between radiographic findings and pPV invasion in patients with PC who underwent resection following NAT, with a particular focus on whether this relationship differs according to the type of NAT administered (NAC or NACRT).

## 2 Materials and Methods

### 2.1 Patients and Eligibility Criteria

We retrospectively collected data from consecutive patients with PC who underwent pancreatectomy after NAT at a single academic educational institution, excluding those with intrapapillary mucinous carcinoma (IPMC), and reviewed their medical records. The decision to administer NAT was based on clinical trials or the standard of care available at the time; therefore, NAT was not determined by tumor status. Postoperative adjuvant chemotherapy, such as gemcitabine or S‐1, was routinely administered for 6 months unless contraindicated [16, 17]. Resectability was classified as resectable (R), borderline resectable (BR), or locally advanced unresectable (UR‐LA) according to the NCCN Guidelines (Pancreatic Adenocarcinoma, version 1.2025) [3]. This study was approved by the Institutional Review Board of Osaka University Hospital (IRB No. 24134) and conducted in accordance with the Declaration of Helsinki. The requirement for informed consent was waived by the ethics committee because of the retrospective study design.

### 2.2 Endpoints

The primary endpoint of this study was to investigate the relationship between radiographic findings (details described below) and pPV invasion in patients with PC who underwent resection following NAT according to the type of NAT (i.e., NAC vs. NACRT). pPV invasion was defined as histological evidence of PV‐SMV invasion in patients who underwent combined PVR. In addition, we investigated the relationship between radiographic findings and the depth of pathological portal vein wall invasion in the NAC and NACRT groups. Survival outcomes were analyzed as supportive data to provide clinical context.

### 2.3 Measuring Method of the Radiographic Degree of PV‐SMV Involvement on CT Imaging

Radiographic PV‐SMV involvement was defined as solid soft‐tissue contact between the tumor and the PV‐SMV on contrast‐enhanced CT, in accordance with the NCCN guidelines, and hazy perivascular attenuation without a definable soft‐tissue interface was not considered to represent PV‐SMV involvement [3]. The PV‐SMV contact length was defined as the maximum contact length between the tumor and the PV‐SMV, and the PV‐SMV contact angle was classified as 0°–360° on the axial image showing the greatest extent of contact. PV‐SMV patency was categorized into three groups: no stenosis, defined as tumor contact without caliber change or disruption on contrast‐enhanced CT; stenosis, defined as luminal narrowing at the site of tumor contact with preserved continuity; and obstruction, defined as complete interruption of portal vein continuity at the site of tumor involvement. The contact angle was evaluated as the circumferential degree of the tumor‐vessel interface on axial CT images and was assessed independently of tumor size or patient body habitus. Radiologic findings were initially assessed by board‐certified diagnostic radiologist and were subsequently re‐evaluated independently by two investigators (N.S. and H.T.) The RECIST classification [18] was used to evaluate the effects of NAT. All indices were measured using dynamic CT performed before and after NAT. Pre‐NAT CT imaging was performed within 1 month before NAT initiation, and post‐NAT CT imaging was performed within 1 month after NAT and before surgery.

### 2.4 Histopathological Assessment

The resected specimens were fixed in 10% formalin for 24 h. They were serially sectioned at 4–5 mm intervals and subjected to histopathological analysis. Histopathological findings were reassessed according to the UICC 8^th^ edition [19], and the Evans classification [20] was applied to evaluate the pathological efficacy of NAT. R0 resection was defined as the absence of tumor cells within 1 mm of any resection margin (margin clearance ≥ 1 mm). All histopathological evaluations were performed by board‐certified pathologists.

### 2.5 Association Between Radiographic Findings and pPV Invasion According to Treatment Modality

The baseline clinical characteristics and radiographic findings of the NAC and NACRT groups are summarized in Table 1. The pPV invasion rate was evaluated within each radiographic subgroup, and its relationship was assessed according to treatment modality (Table 2). The characteristics of patients who underwent PVR and the pPV invasion rate by radiographic subgroup are shown in Tables S1 and S2. The pathological depth of PV wall invasion was classified according to Nakao et al.: [11] grade 0, no invasion; grade 1, tunica adventitia invasion; grade 2, tunica media invasion; and grade 3, tunica intima invasion. The pathological depth of PV wall invasion was then compared across the radiographic subgroups (Table 3).

**TABLE 1:** Patients' characteristics.

	All (*n* = 108)	NAC (*n* = 47)	NACRT (*n* = 61)	*p‐*value
Clinical factors				
Age, median [min–max], years	68 [41–87]	68 [41–82]	68 [41–87]	0.325
Sex, male, %	59 (55%)	21 (45%)	38 (62%)	*0.068*
Pre‐NAT resectability [3], R/BR/UR, %	41/56/11 (38/52/10%)	22/19/6 (47/40/13%)	19/37/5 (31/61/8%)	0.112
Pre‐NAT CA19‐9, median [min–max], U/mL	132 [0.4–14865]	178 [0.4–14865]	1115 [0.4–6621]	0.505
Post‐NAT CA19‐9, median [min–max], U/mL	37.8 [0.4–2897]	33.1 [0.4–2897]	40 [0.4–1111]	0.383
Pre‐NAT tumor size, median [min–max], mm	23 [0–63]	23 [8.5–63]	23 [0–50]	0.874
Post‐NAT tumor size, median [min–max], mm	20 [0–40]	18 [4–40]	20 [0–40]	0.589
Degree of PV‐SMV involvement				
Pre‐NAT PV‐SMV contact length median [min–max], mm	12 [0–35]	12 [0–35]	14 [0–35]	0.160
Post‐NAT PV‐SMV contact length median [min–max], mm	10 [0–35]	9 [0–31]	10 [0–35]	0.412
Pre‐NAT PV‐SMV contact angle < 180°, %	51 (47%)	25 (53%)	26 (43%)	0.275
Post‐NAT PV‐SMV contact angle < 180°, %	72 (67%)	37 (79%)	35 (57%)	***0.018***
PV‐SMV contact angle shrinkage, +, %	51 (47%)	27 (57%)	24 (39%)	*0.061*
Pre‐NAT PV‐SMV stenosis or obstruction, +, %	44 (41%)	14 (30%)	30 (49%)	***0.041***
Post‐NAT PV‐SMV stenosis or obstruction, +, %	39 (36%)	14 (30%)	25 (41%)	0.228
Surgical factors				
Portal vein resection, +, %	67 (62%)	27 (57%)	40 (66%)	0.389
Pathological factors				
Portal vein invasion, +, %	16 (15%)	8 (17%)	8 (13%)	0.790
R0 resection, +, %	97 (91%)	41 (89%)	56 (92%)	0.640
R1 resection at portal notch, +, %	1 (1%)	0	1 (1.6%)	0.284

*Note:* The values shown in italic indicate *p* < 0.10 and the values shown in bold italic indicate *p* < 0.05.

Abbreviations: BR, borderline resectable; CA19‐9, carbohydrate antigen 19‐9; NAC, neoadjuvant chemotherapy; NACRT, neoadjuvant chemoradiotherapy; NAT, neoadjuvant treatment; PD, pancreaticoduodenectomy; PV‐SMV, portal vein‐ superior mesenteric vein; R, resectable; UR, unresectable.

**TABLE 2:** Association between radiographic findings and pPV invasion according to treatment modality.

	All patients (*n* = 108)	NAC (*n* = 47)	NACRT (*n* = 61)	Odds ratio	*p‐*value
pPV invasion rate, %	16 (15%)	8 (17%)	8 (13%)		
Pre‐NAT tumor size					
≥ 20 mm (*n* = 76)	12 (16%)	4/33 (12%)	8/43 (19%)	0.60 [0.16–2.2]	0.438
< 20 mm (*n* = 32)	4 (13%)	4/14 (29%)	0/18 (0%)	—	***0.007***
Pre‐NAT PV‐SMV contact length					
≥ 10 mm (*n* = 83)	12 (14%)	5/34 (15%)	7/48 (16%)	1.01 [0.29–3.5]	0.988
< 10 mm (*n* = 25)	4 (15%)	3/13 (23%)	1/13 (7.7%)	3.6 [0.32–40]	0.267
Pre‐NAT PV‐SMV contact angle					
≥ 180° (*n* = 57)	9 (16%)	3/22 (14%)	6/35 (17%)	0.76 [0.17–3.4]	0.722
< 180° (*n* = 51)	7 (14%)	5/25 (20%)	2/26 (7.7%)	3.0 [0.52–17]	0.196
Pre‐NAT PV‐SMV patency					
Stenosis or obstruction (*n* = 44)	7 (16%)	3/14 (21%)	4/30 (13%)	1.8 [0.34–9.3]	0.503
No stenosis (*n* = 64)	9 (14%)	5/33 (15%)	4/31 (13%)	1.2 [0.29–5.0]	0.796
Post‐NAT tumor size					
≥ 20 mm (*n* = 56)	11 (20%)	4/22 (18%)	7 (21%)	0.85 [0.22–3.3]	0.824
< 20 mm (*n* = 52)	5 (10%)	4/25 (16%)	1 (3.7%)	5.0 [0.51–48]	0.123
Post‐NAT PV‐SMV contact length					
≥ 10 mm (*n* = 58)	13 (22%)	6/22 (27%)	7/36 (19%)	1.6 [0.45–5.2]	0.491
< 10 mm (*n* = 50)	3 (6%)	2/25 (8%)	1/25 (4%)	2.2 [0.18–26]	0.524
Post‐NAT PV‐SMV contact angle					
≥ 180° (*n* = 36)	7 (19%)	2/10 (20%)	5/26 (19%)	1.1 [0.17–6.6]	0.958
< 180° (*n* = 72)	9 (13%)	6/37 (16%)	3/35 (8.6%)	2.1 [0.47–9.0]	0.322
PV‐SMV contact angle shrinkage					
+ (*n* = 51)	4 (7.8%)	3/27 (11%)	1/24 (8.3%)	2.9 [0.28–29]	0.345
− (*n* = 57)	12 (21%)	5/20 (25%)	7/37 (19%)	1.4 [0.38–5.3]	0.594
Post‐NAT PV‐SMV patency					
Stenosis or obstruction (*n* = 39)	8 (21%)	4/27 (29%)	4/25 (16%)	2.1 [0.43–10]	0.358
No stenosis (*n* = 69)	8 (12%)	4/20 (12%)	4/36 (11%)	1.1 [0.25–4.8]	0.896
RECIST classification [18]					
SD or PD (*n* = 79)	11 (14%)	5/32 (16%)	6/47 (13%)	1.3 [0.35–4.6]	0.720
PR (*n* = 29)	5 (17%)	3/15 (20%)	2/14 (14%)	1.5 [0.21–10]	0.683

*Note:* The values shown in italic indicate *p* < 0.10 and the values shown in bold italic indicate *p* < 0.05.

Abbreviations: NAC, neoadjuvant chemotherapy; NACRT, neoadjuvant chemoradiotherapy; NAT, neoadjuvant treatment; PD, progression disease; PR, partial response; SD, stable disease.

**TABLE 3:** Association between radiographic findings and pathological PV‐SMV wall invasion according to treatment modality.

	NAC (*n* = 27)	NACRT (*n* = 38)	*p*‐value
Pathological degree of tumor invasion into PV wall, grade 0/1/2/3, %	19/0/6/2 (70/0/23/7%)	32/2/2/2 (80/5/5/5%)	*0.096*
Post‐NAT tumor size			
≥ 20 mm (*n* = 33)	9/0/4/0	15/2/1/2	*0.058*
< 20 mm (*n* = 32)	10/0/2/2	17/0/1/0	0.108
Post‐NAT PV‐SMV contact length			
≥ 10 mm (*n* = 45)	13/0/5/1	21/2/2/1	0.179
< 10 mm (*n* = 20)	6/0/1/1	11/0/0/1	0.396
Post‐NAT PV‐SMV contact angle			
≥ 180° (*n* = 27)	7/0/2/0	15/0/2/1	0.521
< 180° (*n* = 38)	12/0/4/2	17/2/0/1	***0.024***
Post‐NAT PV‐SMV patency			
Stenosis or obstruction (*n* = 32)	9/0/3/1	17/1/1/0	0.172
No stenosis (*n* = 33)	10/0/3/1	15/1/1/2	0.376
RECIST classification [18]			
SD or PD (*n* = 44)	12/0/4/1	23/2/1/1	0.115
PR (*n* = 21)	7/0/2/1	9/0/1/1	0.762

*Note:* The values shown in italic indicate *p* < 0.10 and the values shown in bold italic indicate *p* < 0.05.

Abbreviations: NAC, neoadjuvant chemotherapy; NACRT, neoadjuvant chemoradiotherapy; NAT, neoadjuvant treatment; PD, progression disease; PR, partial response; SD, stable disease.

### 2.6 Statistical Analyses

Categorical variables were summarized as frequencies and percentages. Pearson's chi‐square test was used for nominal variables. Continuous variables were expressed as medians with ranges. Comparisons between pre‐ and post‐NAT results were made using the paired *t*‐test, while Wilcoxon's test was applied for continuous variables. As supportive information, we presented prognostic data in Supplement materials. Overall survival (OS) was defined as the time from surgery to death or last follow‐up. Patients alive at the last follow‐up were censored at that date. Survival analyses were conducted using the Kaplan–Meier method and compared with a stratified log‐rank test. Statistical significance was set at *p* < 0.05. All analyses were performed using JMP Pro software version 14.0.0 (SAS Institute, Cary, NC, USA).

## 3 Results

### 3.1 Characteristics of All Patients

We identified 272 patients with PC who underwent pancreatectomy after NAT. Of these, 108 patients (40%) with tumors in contact with the PV‐SMV on pre‐ or post‐NAT CT and with an observation period of at least 24 months after resection were included in the study. The characteristics of these patients are shown in Table 1. A total of 47 patients (44%) received NAC, while 61 patients (56%) received NACRT. The NAC regimens included FOLFIRINOX (*n* = 6), gemcitabine + nab‐paclitaxel (*n* = 26), gemcitabine + S‐1 (*n* = 14), and gemcitabine alone (*n* = 1). The NACRT regimens included gemcitabine + S‐1 (*n* = 48), gemcitabine alone (*n* = 10), S‐1 alone (*n* = 2), and gemcitabine + nab‐paclitaxel (*n* = 1). The total radiotherapy dose was 50.4 Gy in all NACRT cases. The pre‐NAT resectability status was 41 (38%), 56 (52%), and 11 (10%) in the R, BR, and UR‐LA groups, respectively. Pre‐NAT CA19‐9 values were significantly higher than post‐NAT CA19‐9 values (pre‐NAT vs. post‐NAT: 132 [0.4–14865] vs. 37.8 [0.4–2897], *p* = 0.001). The R0 resection rate was 91% (97/108). A total of 67 patients (62%) underwent combined PVR. Among these patients, pPV invasion was histologically confirmed in 16 cases (24%), corresponding to 15% of the entire cohort. One patient without PVR underwent R1 resection at the portal notch.

### 3.2 Baseline Characteristics of the NAC and NACRT Groups

Table 1 summarizes the comparison of patient characteristics in the NAC and NACRT groups. There were no significant differences in age, pre‐NAT resectability, CA19‐9 levels, R0 resection rate, number of patients receiving adjuvant chemotherapy, or recurrence rate between the groups (Table 1). A comparison of the pre‐NAT radiographic degree of PV‐SMV involvement showed no statistically significant differences in PV‐SMV contact length or contact angle between the NAC and NACRT groups (Table 1). In contrast, analysis of the post‐NAT radiographic degree of PV‐SMV involvement revealed that a higher proportion of patients in the NAC group had a PV‐SMV contact angle of less than 180° (79% [37/47] vs. 57% [35/61], *p* = 0.018), and there was a trend toward more cases of contact angle shrinkage with NAC (57% [27/47] vs. 39% [24/61], *p* = 0.061). In patients with PVR, pPV invasion was confirmed in 8 patients (17%) in the NAC group and 8 patients (13%) in the NACRT group. In patients without PVR, there were no NAC cases, while 1 NACRT patient (1.6%) underwent R1 resection at the portal notch.

### 3.3 Radiographic Changes After NAT

**FIGURE 1:**
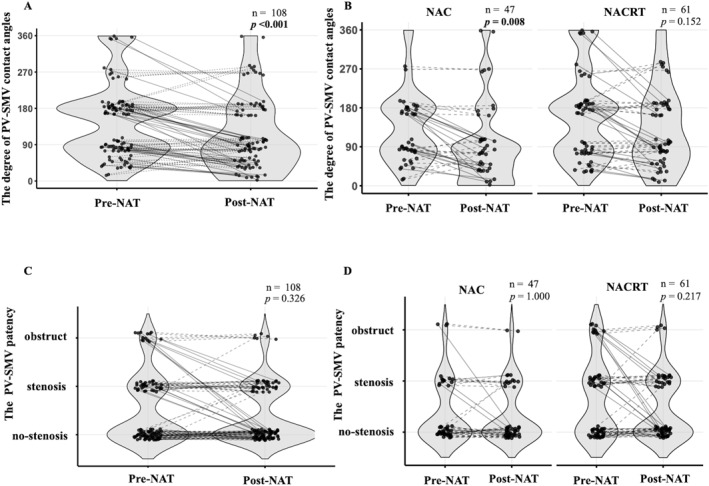
Radiographic changes in PV‐SMV contact angle and PV‐SMV patency from pre‐NAT to post‐NAT in all NAT patients, in the NAC group, and in the NACRT group. Comparison of the radiographic degree of PV‐SMV contact angle between pre‐NAT and post‐NAT imaging in all NAT patients (A), in the NAC group and the NACRT group (B). Comparison of the radiographic degree of PV‐SMV patency between pre‐NAT and post‐NAT imaging in all NAT patients (C), in the NAC group and the NACRT group (D). The Wilcoxon signed‐rank test was used for these analyses. Cases demonstrating a decrease in PV‐SMV contact angle or improvement in PV‐SMV patency from pre‐NAT to post‐NAT were indicated by solid lines, while cases exhibiting no change or deterioration were indicated by dotted lines.

Table 1 and Figure 1 summarize changes in the radiographic findings between pre‐ and post‐NAT assessments. Tumor size and PV‐SMV contact length were significantly reduced after NAT (pre‐NAT vs. post‐NAT: tumor size, 23 [0–63] vs. 20 [0–40], *p* < 0.001; PV‐SMV contact length, 12 [0–35] vs. 10 [0–35], *p* < 0.001). The PV‐SMV contact angle demonstrated a significant reduction from pre‐ to post‐NAT (paired ordinal analysis, *p* < 0.001) (Figure 1A). A significant reduction in the PV‐SMV contact angle was observed within the NAC group (*p* = 0.008), whereas no significant change was observed within the NACRT group (*p* = 0.152) (Figure 1B). A comparison of PV‐SMV patency pre‐ and post‐NAT revealed no significant differences, which was consistent across both the NAC and NACRT groups (Figure 1C,D).

### 3.4 Association Between Radiographic Findings and Pathological PV‐SMV Invasion According to Treatment Modality

pPV invasion was defined as histologically confirmed venous wall invasion in patients who underwent PVR, and the pPV invasion rates in each radiographic subgroup are shown in Table 2. In the subgroup with a pre‐NAT tumor size < 20 mm, the pPV invasion rate was significantly lower in the NACRT group compared with the NAC group (29% [4/14] vs. 0% [0/18], *p* = 0.007; Table 2). Regarding the pre‐NAT radiographic degree of PV‐SMV involvement, no significant difference in pPV invasion rates was observed between the NAC and NACRT groups across subgroups (Table 2). Similarly, no significant differences were found in pPV invasion rates between the groups across post‐NAT radiographic subgroups (Table 2). Analyses in which the single non‐PVR case with an R1 margin at the portal notch was classified as pathologically PV invasion‐positive are provided in Table S3, which shows results consistent with the primary analysis (Table 2).

In patients with PVR (*n* = 67), pPV invasion was observed in 16 patients (24%), 14 of whom could be reassessed for the depth of pPV wall invasion. Two patients were excluded because reassessment of invasion depth was not possible, resulting in 65 patients being included in Table 3. The depth of pPV wall invasion tended to be milder in the NACRT group than in the NAC group (*p* = 0.096) (Table 3). In subgroup analyses, the depth of invasion was significantly milder in NACRT patients than in NAC patients when the post‐NAT PV‐SMV contact angle was < 180° (Table 3).

### 3.5 Survival Analyses

Survival analyses are presented in the (Figures [Link], [Link], [Link]) for descriptive purposes. No clear association between radiographic PV‐SMV involvement and survival outcomes was observed in the present cohort.

## 4 Discussion

In the era of upfront PC surgery, the radiographic degree of PV‐SMV involvement was considered a useful predictor of pPV invasion [11, 15]. In contrast, in the present study limited to patients who received NAT for PC, the radiographic findings showed a limited association with pPV invasion (Table 2). These results indicate that the predictive value of preoperative imaging for venous wall invasion differs substantially between upfront surgery and the NAT setting. Previous reports and NCCN Guidelines have noted the difficulty in predicting the extent of residual viable tumor based on radiographic findings after NAT for PC [3, 4]. The results of our study are consistent with these findings. Even among patients who underwent PVR, the pPV invasion rate was 23% (9/40) in patients with a pre‐NAT PV‐SMV contact angle ≥ 180° and 24% (7/29) in patients with a post‐NAT PV‐SMV contact angle ≥ 180° (Table S2), substantially lower than the 86% reported in patients undergoing upfront surgery [21]. While these results do not allow direct inference regarding treatment efficacy, they indicate that preoperative assessment of pPV invasion in patients who receive NAT remains challenging. One possible explanation is that the NAT‐induced reduction in viable tumor cells is often accompanied by increased fibrosis and fibroinflammatory tissue [22, 23]. This histological change may create a soft‐tissue shadow around the PV‐SMV on radiographic images, resulting in an overestimation of viable tumor contact after NAT, despite actual tumor regression. These mechanisms may help explain our observation that a reduction in PV‐SMV contact angle was more frequently observed in the NAC group than in the NACRT group. However, this apparent radiographic improvement was not accompanied by a corresponding reduction in pPV invasion. This discrepancy suggests that radiographic changes after NAT do not necessarily reflect the histopathological treatment response. Therefore, radiographic change should not be interpreted as a reliable surrogate for pPV invasion, and residual vascular contact on post‐NAT imaging alone should neither dictate intraoperative portal vein management planning nor automatically preclude surgical resection in patients with portal vein involvement considered unresectable on preoperative imaging.

A similar radiologic–pathologic discrepancy has been reported in response assessment after NAT. Miyahara et al. demonstrated that radiologic response evaluated by RECIST criteria was comparable between NAC and NACRT, whereas pathological response was significantly greater after NACRT [4]. Even tumors classified as stable disease on imaging frequently showed marked tumor cell destruction after NACRT. In our cohort, a comparable pattern was observed in the subgroup of patients with pre‐NAT tumor size < 20 mm, in whom pPV invasion was less frequent after NACRT despite persistent radiographic vessel contact. Baseline characteristics, including resectability status (R/BR/UR), were comparable between treatment groups in the small‐tumor subgroup (Table S4), suggesting that the observed difference in pPV invasion was unlikely to be explained solely by baseline imbalance. The difference in pPV invasion rate between NAC and NACRT groups was not observed in patients with tumor size ≥ 20 mm. Reports on other gastrointestinal malignancies have shown that smaller tumor volume predicts a favorable local response to NACRT [24, 25], and our findings are consistent with this evidence. These findings may suggest a greater pathological response to NACRT in patients with smaller tumors.

Survival analyses were performed for descriptive purposes only. Although the degree of radiographic PV‐SMV involvement was strongly associated with prognostic outcomes in patients who underwent upfront surgery for PC [11, 15], no clear association between radiographic PV‐SMV involvement and survival outcomes was observed in the present cohort. (Figures [Link], [Link], [Link]) However, these findings should be interpreted cautiously because the study population represents a selected surgical cohort. Patients who experienced disease progression or distant metastasis during NAT and did not undergo subsequent resection were not included, and therefore the results cannot be generalized to all patients undergoing NAT. For this reason, survival analysis in the present cohort is inherently subject to selection bias and should be interpreted as descriptive rather than as a definitive evaluation of prognostic impact. Accordingly, the present study was not designed to evaluate the prognostic impact of radiographic vascular involvement.

This study had several limitations that could have influenced the results. First, the analysis focused on patients with PC who had radiographic tumor contact with the PV‐SMV before or after NAT and examined them in separate subgroups, leading to small sample sizes in each subgroup and potential bias. Second, because of the retrospective design, NAT regimens varied over time, which may have introduced heterogeneity in patient backgrounds and treatment selection. In addition, because the study included only patients who ultimately underwent resection, the findings should be interpreted as applicable to surgically treated patients after NAT rather than to the entire population of patients receiving NAT. Therefore, this cohort was not designed to evaluate survival outcomes or the prognostic impact of treatment modality. Consequently, larger studies with more uniform patient cohorts are needed to validate these findings.

## 5 Conclusion

After NAT for PC, radiographic PV‐SMV involvement does not necessarily represent pPV invasion. The relationship between radiographic findings and pPV invasion differed between NAC and NACRT in patients with smaller tumors.

## Author Contributions

**Naoko Sekiguchi:** writing – original draft. **Hidenori Takahashi:** conceptualization, writing – review and editing, supervision. **Hirofumi Akita:** writing – review and editing. **Kazuki Sasaki:** writing – review and editing. **Shinichiro Hasegawa:** writing – review and editing. **Yoshifumi Iwagami:** writing – review and editing. **Daisaku Yamada:** writing – review and editing. **Yoshito Tomimaru:** writing – review and editing. **Tadafumi Asaoka:** writing – review and editing. **Takehiro Noda:** writing – review and editing. **Junzo Shimizu:** writing – review and editing. **Shogo Kobayashi:** writing – review and editing. **Yuichiro Doki:** writing – review and editing. **Hidetoshi Eguchi:** writing – review and editing, supervision.

## Funding

The authors have nothing to report.

## Ethics Statement

The requirement for informed consent was waived by the Institutional Review Board Osaka University Hospital (IRB No. 24134) because this was a retrospective study using de‐identified clinical data. All procedures were performed in accordance with the Declaration of Helsinki.

## Conflicts of Interest

The authors declare no conflicts of interest.

## Supporting information


**Figure S1:** Prognostic analyses according to radiographic PV–SMV involvement. Comparison of overall survival (OS) according to radiographic PV–SMV findings on pre‐ and post‐neoadjuvant treatment (NAT) imaging. OS was compared between PV–SMV contact angle < 180° and ≥ 180° on pre‐NAT (A) and post‐NAT (B) images; between PV–SMV contact length < 10 mm and ≥ 10 mm on pre‐NAT (C) and post‐NAT (D) images; and between no stenosis and stenosis/obstruction on pre‐NAT (E) and post‐NAT (F) images.

**Figure S2:** Prognostic analyses according to pre‐neoadjuvant treatment (NAT) radiographic PV–SMV involvement in the NAC and NACRT groups. Comparison of overall survival (OS) according to pre‐NAT radiographic PV–SMV findings in the NAC and NACRT groups. OS was compared between PV–SMV contact angle < 180° and ≥ 180° in the NAC (A) and NACRT (B) groups; between PV–SMV contact length < 10 mm and ≥ 10 mm in the NAC (C) and NACRT (D) groups; and between no stenosis and stenosis/obstruction in the NAC (E) and NACRT (F) groups.

**Figure S3:** Prognostic analyses according to post‐neoadjuvant treatment (NAT) radiographic PV–SMV involvement in the NAC and NACRT groups. Comparison of overall survival (OS) according to post‐NAT radiographic PV–SMV findings in the NAC and NACRT groups. OS was compared between PV–SMV contact angle < 180° and ≥ 180° in the NAC (A) and NACRT (B) groups; between PV–SMV contact length < 10 mm and ≥ 10 mm in the NAC (C) and NACRT (D) groups; and between no stenosis and stenosis/obstruction in the NAC (E) and NACRT (F) groups.

**Table S1:** Patients’ characteristics of patients with PVR.

**Table S2:** Association between radiographic findings and pPV invasion rates between the NAC and NACRT groups in patients with PVR.

**Table S3:** Association between radiographic findings and pPV invasion (including non‐PVR case with an R1 margin at the portal notch) according to treatment modality.

**Table S4:** Baseline characteristics of patients with pre‐NAT tumor size < 20 mm (*n* = 32) according to treatment modality (NAC vs NACRT).

## Data Availability

The data that support the findings of this study are available on request from the corresponding author. The data are not publicly available due to privacy or ethical restrictions.
