# Supplementary figures and images for: Relationship Between Radiographic and Pathological Portal Vein‐Superior Mesenteric Vein Involvement in Neoadjuvant Treatment for Pancreatic Cancer: A Comparative Study of Neoadjuvant Chemotherapy and Chemoradiotherapy
Source: World J Surg. 2026 May 7;50(6):1676–84. doi: 10.1002/wjs.70395 (PMC13242061; doi:10.1002/wjs.70395)

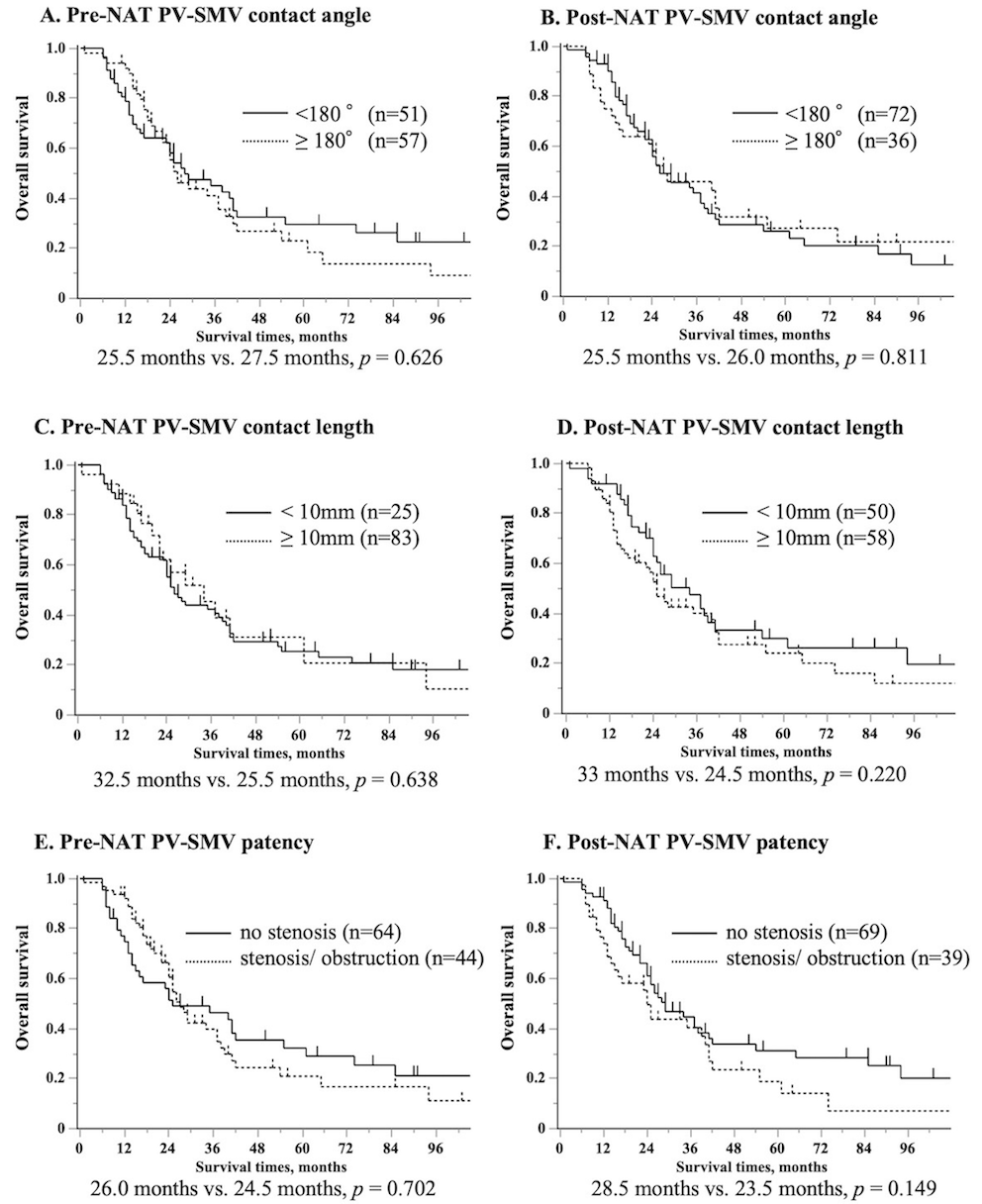

Supplement: Supplementary file 1 — Figure S1: Prognostic analyses according to radiographic PV–SMV involvement. Comparison of overall survival (OS) according to radiographic PV–SMV findings on pre‐ and post‐neoadjuvant treatment (NAT) imaging. OS was compared between PV–SMV contact angle < 180° and ≥ 180° on pre‐NAT (A) and post‐NAT (B) images; between PV–SMV contact length < 10 mm and ≥ 10 mm on pre‐NAT (C) and post‐NAT (D) images; and between no stenosis and stenosis/obstruction on pre‐NAT (E) and post‐NAT (F) images. [file WJS-50-1676-s006.tiff]

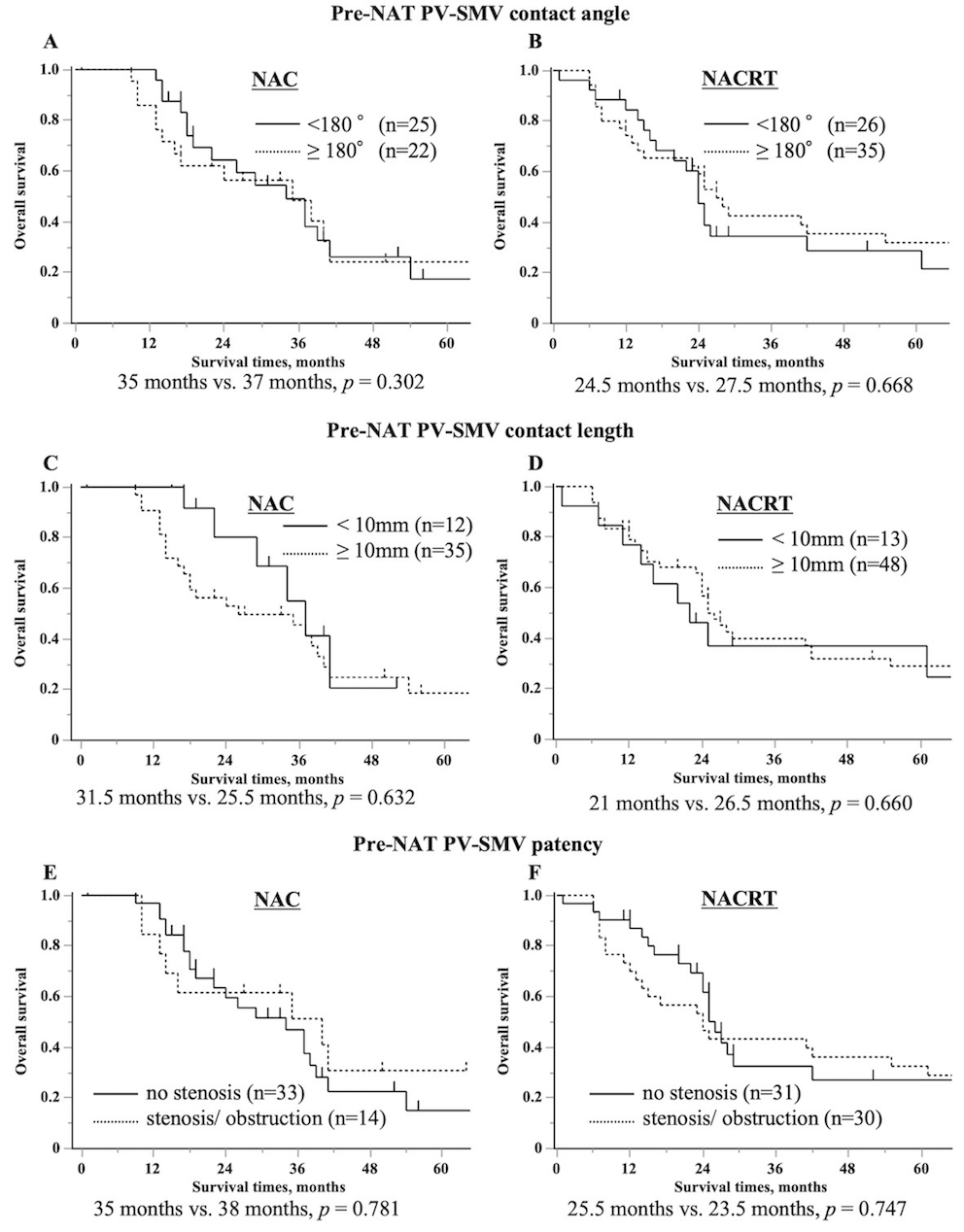

Supplement: Supplementary file 2 — Figure S2: Prognostic analyses according to pre‐neoadjuvant treatment (NAT) radiographic PV–SMV involvement in the NAC and NACRT groups. Comparison of overall survival (OS) according to pre‐NAT radiographic PV–SMV findings in the NAC and NACRT groups. OS was compared between PV–SMV contact angle < 180° and ≥ 180° in the NAC (A) and NACRT (B) groups; between PV–SMV contact length < 10 mm and ≥ 10 mm in the NAC (C) and NACRT (D) groups; and between no stenosis and stenosis/obstruction in the NAC (E) and NACRT (F) groups. [file WJS-50-1676-s004.tiff]

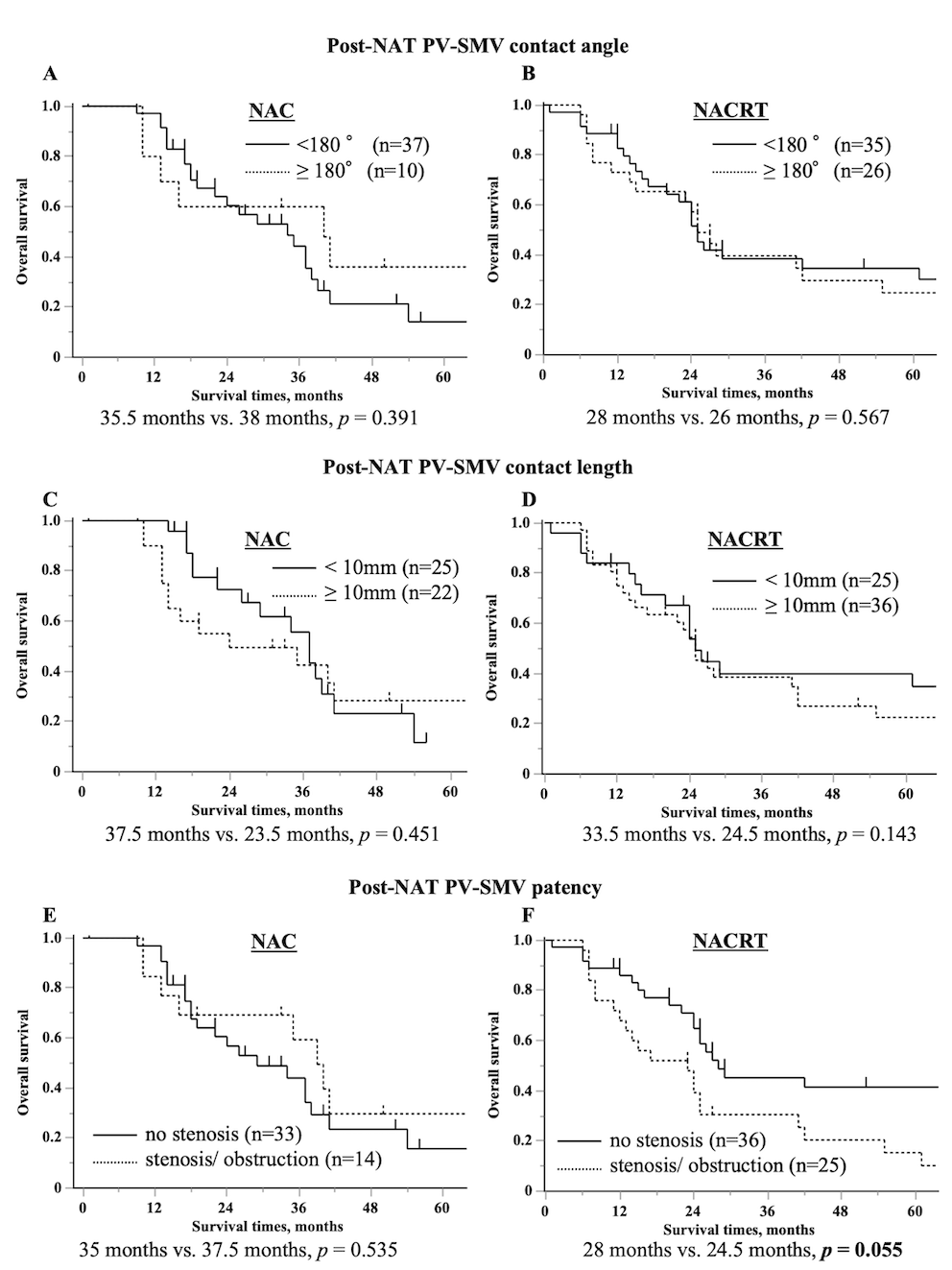

Supplement: Supplementary file 3 — Figure S3: Prognostic analyses according to post‐neoadjuvant treatment (NAT) radiographic PV–SMV involvement in the NAC and NACRT groups. Comparison of overall survival (OS) according to post‐NAT radiographic PV–SMV findings in the NAC and NACRT groups. OS was compared between PV–SMV contact angle < 180° and ≥ 180° in the NAC (A) and NACRT (B) groups; between PV–SMV contact length < 10 mm and ≥ 10 mm in the NAC (C) and NACRT (D) groups; and between no stenosis and stenosis/obstruction in the NAC (E) and NACRT (F) groups. [file WJS-50-1676-s002.tiff]
