# Supplementary material for: Relationship Between Radiographic and Pathological Portal Vein‐Superior Mesenteric Vein Involvement in Neoadjuvant Treatment for Pancreatic Cancer: A Comparative Study of Neoadjuvant Chemotherapy and Chemoradiotherapy
Source: World J Surg. 2026 May 7;50(6):1676–84. doi: 10.1002/wjs.70395 (PMC13242061; doi:10.1002/wjs.70395)
Supplement: Supplementary file 4 — Table S1: Patients’ characteristics of patients with PVR. [file WJS-50-1676-s003.docx]

**Supplementary Table 1: Patients’ characteristics of patients with PVR**

|  | **All**  **(n = 67)** | **NAC**  **(n = 27)** | **NACRT**  **(n = 40)** | ***p*-value** |
| --- | --- | --- | --- | --- |
| **Clinical factors** |  |  |  |  |
| Age, median[min-max], years | 68[41–87] | 69[43–82] | 68[41–87] | 0.772 |
| Sex, male, % | 34(51%) | 12(44%) | 22(55%) | 0.396 |
| Pre-NAT resectability ^3^, R/BR/UR, % | 19/42/6 (28%/63%/9%) | 11/13/3  (41%/48%/11%) | 8/29/3  (20%/73%/7.5%) | 0.123 |
| Pre-NAT CA19-9, median[min-max], U/ml | 210[0.4–14865] | 300[0.4–14865] | 154[2.5–6621] | 0.160 |
| Post-NAT CA19-9, median[min-max], U/ml | 43.5[0.4–2897] | 40.5[0.4–2897] | 48.9[2–1111] | 0.930 |
| Adjuvant chemotherapy, +, % |  |  |  |  |
| Pre-NAT tumor size, median[min-max], mm | 25[10–63] | 25[10–63] | 24[10–50] | 0.883 |
| Post-NAT tumor size, median[min-max], mm | 20[5–40] | 18[5–40] | 20[7–35] | 0.944 |
| **Degree of PV-SMV involvement** |  |  |  |  |
| Pre-NAT PV-SMV contact length  median[min-max], mm | 15[0–35] | 14[0–35] | 15[0–33] | 0.793 |
| Post-NAT PV-SMV contact length  median[min-max], mm | 11[0–35] | 11[0–31] | 10[0–35] | 0.880 |
| Pre-NAT PV-SMV contact angle <180°, % | 27(40%) | 11(41%) | 16(40%) | 0.952 |
| Post-NAT PV-SMV contact angle <180°, % | 38(57%) | 18(67%) | 20(50%) | 0.174 |
| PV-SMV contact angle shrinkage, +, % | 26(39%) | 13(48%) | 13(33%) | 0.198 |
| Pre-NAT PV-SMV stenosis or obstruction  +, % | 35(52%) | 12(44%) | 23(58%) | 0.294 |
| Post-NAT PV-SMV stenosis or obstruction  +, % | 33(49%) | 13(48%) | 20(50%) | 0.882 |
| **Surgical factors** |  |  |  |  |
| Surgical method  Pancreaticoduodenectomy, % | 61(91%) | 24(89%) | 37(93%) | 0.615 |
| Operation time, median[min-max], min | 543[292–831] | 539[317–831] | 534[292–816] | 0.306 |
| Blood loss, median[min-max], ml | 710[170–3980] | 695[240–1970] | 730[170–3980] | 0.943 |
| **Pathological factors** |  |  |  |  |
| Pathological tumor size  median[min-max], mm | 22[0–55] | 24[0–40] | 21.5[3–55] | 0.398 |
| Extension beyond the pancreas, +, % | 60(92%) | 24(92%) | 36(92%) | 1.000 |
| PV invasion, +, % | 16(24%) | 8(30%) | 8(20%) | 0.368 |
| Pathological degree of tumor invasion into PV wall, Grade 0/1/2/3 | 51/2/9/3  (78/3/14/4%) | 19/0/6/2  (70/0/22/3.7%) | 32/2/2/2  (80/5/5/5%) | *0.096* |
| Artery invasion, +, % | 3(4.7%) | 1(3.9%) | 2(5.3%) | 0.790 |
| Degree of differentiation, poorly differentiated, +, % | 5(7.5%) | 2(7.4%) | 3(7.5%) | 0.989 |
| Peritumoral lymphatic or venous invasion  +, % | 30(46%) | 13(50%) | 17(44%) | 0.612 |
| Perineural invasion, +, % | 56(86%) | 23(88%) | 33(85%) | 0.657 |
| Lymphatic metastasis, +, % | 33(51%) | 19(73%) | 14(36%) | ***0.003*** |
| R0 resection, +, % | 59(89%) | 21(81%) | 38(95%) | *0.069* |
| Evans classification ^20^, ≥ IIb, % | 12(18%) | 4(15%) | 8(20%) | 0.390 |
| **Oncological outcomes** |  |  |  |  |
| Recurrence, +, % | 51(76%) | 23(88%) | 28(70%) | *0.070* |
| Isolated local recurrence, % | 14(27%) | 11(48%) | 3(11%) | ***0.010*** |
| Distant metastasis, % | 29(57%) | 9(39%) | 20(71%) |  |
| Local and distant metastasis, % | 8(16%) | 3(13%) | 5(18%) |  |
| Death, +, % | 48(72%) | 18(67%) | 30(75%) | 0.391 |

**Abbreviations:** PVR, portal vein resection; NAT, neoadjuvant treatment; NAC, neoadjuvant chemotherapy; NACRT, neoadjuvant chemoradiotherapy; PV-SMV, portal vein- superior mesenteric vein; CA19-9, carbohydrate antigen 19-9; R, resectable; BR. borderline resectable; UR, unresectable
