# Supplementary material for: Relationship Between Radiographic and Pathological Portal Vein‐Superior Mesenteric Vein Involvement in Neoadjuvant Treatment for Pancreatic Cancer: A Comparative Study of Neoadjuvant Chemotherapy and Chemoradiotherapy
Source: World J Surg. 2026 May 7;50(6):1676–84. doi: 10.1002/wjs.70395 (PMC13242061; doi:10.1002/wjs.70395)
Supplement: Supplementary file 5 — Table S2: Association between radiographic findings and pPV invasion rates between the NAC and NACRT groups in patients with PVR. [file WJS-50-1676-s005.docx]

**Supplementary Table 2: Association between radiographic findings and pPV invasion rates between the NAC and NACRT groups in patients with PVR**

|  | **NAC (n=27)** | **NACRT (n=40)** | **Odds ratio** | ***p*-value** |
| --- | --- | --- | --- | --- |
| **pPV invasion rate, %** | **8 (30%)** | **8 (20%)** |  | **0.368** |
| **Pre-NAT tumor size** |  |  |  |  |
| ≥ 20 mm (n = 52) | 4/20 (20%) | 8/32 (28%) | 0.75[0.19–2.9] | 0.675 |
| < 20 mm (n = 15) | 4/7 (57%) | 0 | - | ***0.005*** |
| **Pre-NAT PV-SMV contact length** |  |  |  |  |
| ≥ 10 mm (n = 54) | 5/23 (22%) | 7/31 (23%) | 0.95[0.26–3.5] | 0.941 |
| < 10 mm (n = 13) | 3/4 (75%) | 1/9 (11%) | 24[1.1–518] | ***0.022*** |
| **Pre-NAT PV-SMV contact angle** |  |  |  |  |
| ≥ 180 ° (n = 40) | 3/16 (19%) | 6/24 (25%) | 0.69[0.15–3.3] | 0.640 |
| < 180 ° (n = 27) | 5/11 (45%) | 2/16(17%) | 5.8[0.87–39] | 0.055 |
| **Pre-NAT PV-SMV patency** |  |  |  |  |
| Stenosis or obstruction (n = 35) | 3/12 (25%) | 4/23 (17%) | 1.6[0.29–8.6] | 0.598 |
| No stenosis (n = 32) | 5/15 (33%) | 4/17 (24%) | 1.6[0.34–7.7] | 0.539 |
| **Post-NAT tumor size** |  |  |  |  |
| ≥ 20 mm (n = 35) | 4/13 (31%) | 7/22 (32%) | 0.95[0.22–4.2] | 0.949 |
| < 20 mm (n = 32) | 4/14 (29%) | 1/18 (5.6%) | 6.8[0.66–70] | *0.071* |
| **Post-NAT PV-SMV contact length** |  |  |  |  |
| ≥ 10 mm (n = 46) | 6/18 (33%) | 7/28 (25%) | 1.4[0.38–5.0] | 0.622 |
| < 10 mm (n = 21) | 2/9 (22%) | 1/12 (8.3%) | 3.1[0.23–42] | 0.369 |
| **Post-NAT PV-SMV contact angle** |  |  |  |  |
| ≥ 180 ° (n = 29) | 2/9 (22%) | 5/20 (25%) | 0.86[0.13–5.6] | 0.871 |
| < 180 ° (n = 38) | 6/18 (33%) | 3/20 (15%) | 2.8[0.59–14] | 0.182 |
| **PV-SMV contact angle shrinkage** |  |  |  |  |
| + (n = 25) | 3/13 (23%) | 1/12 (8.3%) | 3.3[0.23–7] | 0.305 |
| – (n = 42) | 5/14 (36%) | 7/28 (25%) | 1.6[0.39–6.4] | 0.517 |
| **Post-NAT PV-SMV patency** |  |  |  |  |
| Stenosis or obstruction (n = 33) | 4/13 (31%) | 4/20 (20%) | 1.8[0.36–8.9] | 0.484 |
| No stenosis (n = 34) | 4/14 (29%) | 4/20 (20%) | 1.6[0.32-7.9] | 0.564 |
| **RECIST classification** ^18^ |  |  |  |  |
| SD or PD (n = 46) | 5/17 (29%) | 6/29 (20%) | 1.6[0.40–6.3] | 0.507 |
| PR (n = 21) | 3/10 (30%) | 2/11 (18%) | 1.9[0.25–15] | 0.525 |

**Abbreviations:** NAT, neoadjuvant treatment; NAC, neoadjuvant chemotherapy; NACRT, neoadjuvant chemoradiotherapy; PVR, portal vein resection; SD, stable disease; PD, progression disease; PR, partial response
