# Supplementary material for: Relationship Between Radiographic and Pathological Portal Vein‐Superior Mesenteric Vein Involvement in Neoadjuvant Treatment for Pancreatic Cancer: A Comparative Study of Neoadjuvant Chemotherapy and Chemoradiotherapy
Source: World J Surg. 2026 May 7;50(6):1676–84. doi: 10.1002/wjs.70395 (PMC13242061; doi:10.1002/wjs.70395)
Supplement: Supplementary file 6 — Table S3: Association between radiographic findings and pPV invasion (including non‐PVR case with an R1 margin at the portal notch) according to treatment modality. [file WJS-50-1676-s007.docx]

**Supplementary Table 3: Association between radiographic findings and pPV invasion (including non-PVR case with an R1 margin at the portal notch) according to treatment modality**

|  | **All patients (n = 108)** | ***p-*value** | **NAC**  **(n = 47)** | **NACRT**  **(n = 61)** | **Odds ratio** | ***p-*value** |
| --- | --- | --- | --- | --- | --- | --- |
| **pPV invasion rate, %** | 17 (16%) |  | 8 (17%) | 9 (15%) | 1.2[0.42–3.3] | 0.723 |
| **Pre-NAT tumor size** | | | | | | |
| ≥ 20 mm (n = 76) | 13 (17%) | 0.541 | 4/33 (12%) | 9/43 (21%) | 0.52[0.14–1.9] | 0.305 |
| < 20 mm (n = 32) | 4 (13%) |  | 4/14 (29%) | 0/18 (0%) | - | **0.007** |
| **Pre-NAT PV-SMV contact length** | | | | | | |
| ≥ 10 mm (n = 83) | 13 (16%) | 0.954 | 5/34 (15%) | 8/48 (17%) | 0.86[0.25–2.9] | 0.810 |
| < 10 mm (n = 25) | 4 (15%) |  | 3/13 (23%) | 1/13 (7.7%) | 3.6[0.32–40] | 0.267 |
| **Pre-NAT PV-SMV contact angle** | | | | | | |
| ≥ 180 ° (n = 57) | 10 (18%) | 0.585 | 3/22 (14%) | 7/35 (20%) | 0.63[0.14–2.8] | 0.533 |
| < 180 ° (n = 51) | 7 (14%) |  | 5/25 (20%) | 2/26 (7.7%) | 3.0[0.52–17] | 0.196 |
| **Pre-NAT PV-SMV patency** | | | | | | |
| Stenosis or obstruction (n = 44) | 8 (18%) | 0.566 | 3/14 (21%) | 5/30 (17%) | 1.4[0.27–6.7] | 0.706 |
| No stenosis (n = 64) | 9 (14%) |  | 5/33 (15%) | 4/31 (13%) | 1.2[0.29–5.0] | 0.796 |
| **Post-NAT tumor size** | | | | | | |
| ≥ 20 mm (n = 56) | 11 (20%) | 0.244 | 4/22 (18%) | 7 (21%) | 0.85[0.22–3.3] | 0.824 |
| < 20 mm (n = 52) | 6 (12%) |  | 4/25 (16%) | 2 (7.4%) | 2.3[0.40–14] | 0.330 |
| **Post-NAT PV-SMV contact length** | | | | | | |
| ≥ 10 mm (n = 58) | 14 (24%) | 0.008 | 6/22 (27%) | 8/36 (22%) | 1.3[0.38–4.5] | 0.664 |
| < 10 mm (n = 50) | 3 (6%) |  | 2/25 (8%) | 1/25 (4%) | 2.2[0.18–26] | 0.524 |
| **Post-NAT PV-SMV contact angle** | | | | | | |
| ≥ 180 ° (n = 36) | 8 (22%) | 0.200 | 2/10 (20%) | 6/26 (23%) | 0.83[0.14–5.0] | 0.841 |
| < 180 ° (n = 72) | 9 (13%) |  | 6/37 (16%) | 3/35 (8.6%) | 2.1[0.47–9.0] | 0.322 |
| **PV-SMV contact angle shrinkage** | | | | | | |
| + (n = 51) | 12 (21%) | 0.104 | 3/27 (11%) | 2/24 (8.3%) | 1.4[0.21–9.0] | 0.738 |
| – (n = 57) | 5 (9.8%) |  | 5/20 (25%) | 7/37 (19%) | 1.4[0.38–5.3] | 0.594 |
| **Post-NAT PV-SMV patency** | | | | | | |
| Stenosis or obstruction (n = 39) | 9 (23%) | 0.122 | 4/27 (29%) | 5/25 (20%) | 1.6[0.35–7.3] | 0.546 |
| No stenosis (n = 69) | 8 (12%) |  | 4/20 (12%) | 4/36 (11%) | 1.1[0.25–4.8] | 0.896 |
| **RECIST classification**^17^ | | | | | | |
| SD or PD (n = 79) | 11 (14%) | 0.403 | 5/32 (16%) | 6/47 (13%) | 1.3[0.35–4.6] | 0.720 |
| PR (n = 29) | 6 (21%) |  | 3/15 (20%) | 3/14 (21%) | 0.91[0.15–5.5] | 0.924 |

**Abbreviations:** PVR, portal vein resection; NAT, neoadjuvant treatment; NAC, neoadjuvant chemotherapy; NACRT, neoadjuvant chemoradiotherapy; PV-SMV, portal vein- superior mesenteric vein; CA19-9, carbohydrate antigen 19-9; R, resectable; BR. borderline resectable; UR, unresectable; SD, stable disease; PD, progression disease; PR, partial response
