# Supplementary material for: Relationship Between Radiographic and Pathological Portal Vein‐Superior Mesenteric Vein Involvement in Neoadjuvant Treatment for Pancreatic Cancer: A Comparative Study of Neoadjuvant Chemotherapy and Chemoradiotherapy
Source: World J Surg. 2026 May 7;50(6):1676–84. doi: 10.1002/wjs.70395 (PMC13242061; doi:10.1002/wjs.70395)
Supplement: Supplementary file 7 — Table S4: Baseline characteristics of patients with pre‐NAT tumor size < 20 mm (n = 32) according to treatment modality (NAC vs NACRT). [file WJS-50-1676-s001.docx]

**Supplementary Table 4: Baseline characteristics of patients with pre-NAT tumor size < 20 mm　(n=32) according to treatment modality (NAC vs NACRT).**

|  | **NAC**  **(n = 14)** | **NACRT**  **(n = 18)** | ***p*-value** |
| --- | --- | --- | --- |
| **pPV invasion rate, %** | **4 (29%)** | **0 (0%)** | **0.007** |
| **Age, median[min-max], years** | 72.5[53–78] | 68.5[51–82] | 0.865 |
| **Sex, male, %** | 6 (43%) | 10 (56%) | 0.475 |
| **Pre-NAT resectability ^3^, R/BR/UR, %** | 9/3/2 (64/22/14%) | 12/6/0 (67/33/0%) | 0.156 |
| **Pre-NAT CA19-9, median[min-max], U/ml** | 102.4[5–2037] | 37[0.4–688] | 0.280 |
| **Post-NAT CA19-9, median[min-max], U/ml** | 39.9[3.6–2897] | 22[0.4–467] | 0.655 |
| **Pre-NAT PV-SMV contact length** |  |  |  |
| ≥ 10 mm (n = 20) | 7 (50%) | 13 (72%) | 0.197 |
| **Pre-NAT PV-SMV contact angle** |  |  |  |
| ≥ 90 ° (n = 20) | 8 (57%) | 12 (67%) | 0.581 |
| ≥ 180 ° (n = 9) | 3 (21%) | 6 (33%) | 0.454 |
| ≥ 270 ° (n = 3) | 1 (7.1%) | 2 (11%) | 0.699 |
| **Pre-NAT PV-SMV patency** |  |  |  |
| Stenosis or obstruction (n = 7) | 1 (7.1%) | 6 (33%) | 0.061 |

**Abbreviations:** NAT, neoadjuvant treatment; NAC, neoadjuvant chemotherapy; NACRT, neoadjuvant chemoradiotherapy; R, resectable; BR, borderline resectable; UR, unresectable; CA19-9, carbohydrate antigen 19-9; PV-SMV, portal vein- superior mesenteric vein
